# MipLAAO, a new L-amino acid oxidase from the redtail coral snake *Micrurus mipartitus*

**DOI:** 10.7717/peerj.4924

**Published:** 2018-06-08

**Authors:** Paola Rey-Suárez, Cristian Acosta, Uday Torres, Mónica Saldarriaga-Córdoba, Bruno Lomonte, Vitelbina Núñez

**Affiliations:** 1 Programa de Ofidismo y Escorpionismo, Facultad de Ciencias Farmacéuticas y Alimentarias, Universidad de Antioquia, Medellín, Colombia; 2 Centro de Investigación en Recursos Naturales y Sustentabilidad, Universidad Bernardo O’Higgins, Santiago de Chile, Chile; 3 Instituto Clodomiro Picado, Facultad de Microbiología, Universidad de Costa Rica, San José, Costa Rica; 4 Escuela de Microbiología, Universidad de Antioquia, Medellín, Colombia

**Keywords:** Antibacterial activity, L-Amino acid oxidase, *Escherichia coli*, *Staphyloccocus aureus*, *Micrurus mipartitus*, Snake venom, Protein sequence, Coral snake

## Abstract

L-amino acid oxidases (LAAOs) are ubiquitous enzymes in nature. Bioactivities described for these enzymes include apoptosis induction, edema formation, induction or inhibition of platelet aggregation, as well as antiviral, antiparasite, and antibacterial actions. With over 80 species, *Micrurus* snakes are the representatives of the Elapidae family in the New World. Although LAAOs in *Micrurus* venoms have been predicted by venom gland transcriptomic studies and detected in proteomic studies, no enzymes of this kind have been previously purified from their venoms. Earlier proteomic studies revealed that the venom of *M. mipartitus* from Colombia contains ∼4% of LAAO. This enzyme, here named MipLAAO, was isolated and biochemically and functionally characterized. The enzyme is found in monomeric form, with an isotope-averaged molecular mass of 59,100.6 Da, as determined by MALDI-TOF. Its oxidase activity shows substrate preference for hydrophobic amino acids, being optimal at pH 8.0. By nucleotide sequencing of venom gland cDNA of mRNA transcripts obtained from a single snake, six isoforms of MipLAAO with minor variations among them were retrieved. The deduced sequences present a mature chain of 483 amino acids, with a predicted pI of 8.9, and theoretical masses between 55,010.9 and 55,121.0 Da. The difference with experimentally observed mass is likely due to glycosylation, in agreement with the finding of three putative N-glycosylation sites in its amino acid sequence. A phylogenetic analysis of MmipLAAO placed this new enzyme within the clade of homologous proteins from elapid snakes, characterized by the conserved Serine at position 223, in contrast to LAAOs from viperids. MmipLAAO showed a potent bactericidal effect on *S. aureus* (MIC: 2 µg/mL), but not on *E. coli*. The former activity could be of interest to future studies assessing its potential as antimicrobial agent.

## Introduction

L-amino acid oxidases (LAAOs, E.C. 1.4.3.2) are flavoenzymes that catalyze the stereospecific oxidative deamination of L-amino acid substrate to α-keto acid, producing ammonia and hydrogen peroxide (Izidoro et al., 2014). These enzymes are widely distributed in different organisms such as bacteria (Huang et al., 2011; Hossain et al., 2014) fungi (Davis, Askin & Hynes, 2005; Pišlar et al., 2016), birds (Struck & Sizer, 1960) mammals (Puiffe et al., 2013), and plants (Du & Clemetson, 2002). In snake venoms (svLAAO) they are present in the Viperidae and Elapidae families, in amounts between 0.1 and 30% of total protein content (Izidoro et al., 2014). Also, these enzymes have been found in non-venomous snakes such as *Python regius* and *Pantherophis guttatus* (Hargreaves et al., 2014). These proteins are responsible for the characteristic yellow color of many snake venoms (Izidoro et al., 2014).

svLAAOs are generally homodimeric glycoproteins (with approximately 4% of carbohydrates), with molecular masses ranging between 120 and 150 kDa in native forms, and from 55 to 66 kDa in monomeric forms, possibly with a non-covalent association between the two subunits (Du & Clemetson, 2002). They have a wide range of isoelectric points (pI), from about 4.4 to 8.5, and they can bind either to flavine mononucleotide (FMN) or to flavine adenine dinucleotide (FAD) (Izidoro et al., 2014). Most svLAAOs demonstrate a relatively high affinity for hydrophobic and aromatic amino acids, including L-Phe, L-Met, L-Leu and L-Ile because of substrate specificity related to side-chain binding sites (Costa et al., 2014; Geueke & Hummel, 2002), and they are sensitive to temperature, pH changes and lyophilization (Du & Clemetson, 2002).

svLAAOs are multifunctional enzymes exhibiting a wide range of biological activities including apoptosis induction (Tan et al., 2017; Carone et al., 2017; Costa et al., 2017), edema formation (Ali et al., 2000; Teixeira et al., 2016), inhibition or induction of platelet aggregation (Takatsuka et al., 2001; Samel et al., 2008; Naumann et al., 2011), leishmanicidal (Tempone et al., 2001; Ciscotto et al., 2009; Stábeli et al., 2007; Carone et al., 2017)  and antibacterial functions, among other activities.

The antibacterial activity of svLAAOs was reported more than forty years ago (Skarnes, 1970) in *Crotalus adamanteus* venom. Since then, other authors have also reported antibacterial activity against Gram-positive and Gram-negative bacteria (Toyama et al., 2006; Stábeli et al., 2007; Rodrigues et al., 2009; Zhong et al., 2009; Lee et al., 2011; Vargas et al., 2013; Vargas Muñoz et al., 2014; Lazo et al., 2017). However, such activity has not been reported for a *Micrurus mipartitus* venom enzyme. The aim of this work was to isolate and characterize the biochemical properties of LAAO from this venom, and its antibacterial activity.

## Materials and Methods

### Isolation of MipLAAO

MipLAAO, the fraction identified as LAAO in proteomic analysis of *M. mipartitus* venom (Rey-Suárez et al., 2011), was isolated using size exclusion chromatography in a high performance liquid chromatographic system (SEC-HPLC). For this, two mg of lyophilized venom (obtained from specimens maintained in Serpentarium of Universidad de Antioquia from the western region of Antioquia, Colombia), were dissolved in PBS (phosphate-buffered saline; 0.12 M NaCl, 40 mM sodium phosphate, pH 7.2) and separated on a Biosec S-2000 column (Phenomenex, 5 µm particle diameter; 300 × 7.8 mm), using a Shimadzu Prominence-20A chromatograph, monitored at 215 nm. Elution was performed at 0.7 mL/min using the same buffer. Five chromatographic separations were repeated and the LAAO fraction was collected, immediately concentrated, and finally desalted with Amicon Ultra filters (MWCO 10,000 membrane).

To assess the purity of MipLAAO, the protein was submitted to analytical reverse-phase high performance liquid chromatographic (RP-HPLC) using a C_18_ column (Pinnacle, 5 µm particle diameter; 250 × 4.6 mm), eluted at 1 mL/min with a linear gradient from 0 to 70% solution B (0.1% TFA, 99.9% acetonitrile) in 25 min. Electrophoretic homogeneity was evaluated by SDS-PAGE (Laemmli, 1970) after reduction with 5% 2-mercaptoethanol at 100 °C for 5 min.

### Molecular mass determination

The molecular mass of MipLAAO was determined by MALDI-TOF mass spectrometry. The protein (1 µg) was diluted in water-TFA (0.1%), mixed at 1:1 with saturated sinapinic acid in 50% acetonitrile, 0.1% TFA, and spotted (1 µL) onto an OptiToF-384 plate for MALDI-TOF MS analysis. Spectra were acquired on an Applied Biosystems 4800-Plus instrument (Foster City, CA, USA), using 500 shots/spectrum and a laser intensity of 4,200, in linear positive mode, over the m/z range 10,000–80,000.

### Protein identification by MALDI-TOF/TOF peptide sequencing

For MS peptide sequencing, MipLAAO (40 µg) was reduced with dithiothreitol (10 mM), and alkylated with iodoacetamide (50 mM), followed by digestion with sequencing grade trypsin for 24 h at 37 °C, as described (Rey-Suárez et al., 2012). The resulting peptides were separated by RP-HPLC on a C_18_ column (2.1 × 150 mm; Phenomenex), eluted at 0.3 mL/min with a 0–70% acetonitrile gradient over 40 min, manually collected, and dried in a vacuum centrifuge (Vacufuge, Eppendorf). Peptides were redissolved in 50% acetonitrile, 0.1% TFA, and analyzed by MALDI-TOF/TOF (4,800 Plus Proteomics Analyzer; Applied Biosystems, Foster City, CA, USA) using α-cyano-hydroxycinnamic acid as matrix, at 2 kV in positive reflectron mode, 500 shots/spectrum, and a laser intensity of 3,000. Fragmentation spectra were initially searched using the Paragon^®^ algorithm of ProteinPilot 4.0 (AB; Sciex, Framingham, MA, USA) against the UniProt/SwissProt serpentes database, and further they were manually interpreted *de novo*.

### Snake venom gland cDNA synthesis and sequencing

mRNA was extracted from *M. mipartitus* venom gland of a specimen which died in captivity in the Serpentarium of Universidad de Antioquia, using the trizol method (Life Technologies, Carlsbad, CA, USA). The cDNA was obtained using Maxima First Strand cDNA Synthesis Kit (Thermo Fisher Scientific, Waltham, MA, USA). The conditions used in thermocycler were 95 °C for 8 min, followed by 10 min at 95 °C, 10 min at 53 °C and 2 min at 72 °C and 7 min at 72 °C to complete the cycle.

The LAAO-cDNA was obtained using the forward primer 5′-GAT GAA TGT CTT CTT TAT GTT CTC-3′, and the reverse 5′-GCA AGA GAT GTG AAT CGT GCT-3′ and PCR Supermix (Invitrogen), and conditions described by Pierre et al. (2005). The purified product was ligated to the pGEM-T Easy cloning vector (Promega, Madison, WI, USA) and used to transform *Escherichia coli* competent cells DH5-α and TOP10. The cells were cultured on LB (Luria-Bertani) agar, and transformed colonies were used to the obtain the plasmid using the QIA prep Spin Miniprep kit (QIAGEN, Hilden, Germany). The extracted product was sent to Macrogen Korea, specifying that it corresponded to complete plasmids with gene inserted at the multiple cloning site, to be sequenced from the vector promoter T7 and SP6 in order to confirm the direction in which the construct was inserted into the vector.

### Bioinformatics procedures

The edition of cDNA sequences was performed in BioEdit version 7.0 (Hall, 1999). Nucleotide sequences were translated into amino acids to evaluate the reading frame and ensure the absence of premature stop codons or other nonsense mutation using GeneDoc software (Nicholas, Nicholas & Deerfield, 1997). SignalP 4.01 server available at http://www.cbs.dtu.dk/services/SignalP/ was used for signal peptide prediction (Petersen et al., 2011) and the posterior analyses were performed only with the predicted mature protein. NetNglyc (http://www.cbs.dtu.dk/services/NetNGlyc/) was used to predict the glycosylation sites.

### Phylogenetic relationships and genetic distance

Bayesian inference (BI) algorithms implemented in MrBayes v3.0B4 (Ronquist & Huelsenbeck, 2003) was used to infer phylogenetic trees. A total of 48 related amino acid sequences of venom LAAOs, including 17 from Elapidae, 30 from Viperidae and one from Polychrotidae were obtained in two protein databases, BLAST (BLASTp http://blast.ncbi.nlm.nih.gov/Blast.cgi) and Uniprot (http://www.uniprot.org/blast/). These sequences were selected with E-values close to zero and with percentage of identity >30% (Pearson, 2013) (Supplementary material S1).

The multiple alignment of amino acid sequences of mature chains was performed in PRALINE (Heringa, 1999) using default parameters. After including gaps to maximize alignments, the final number of amino acid positions was 500. For phylogenetic analysis we used *Anolis carolinensis* sequence (R4GD21) as outgroup. The best-fitting model of amino acid substitution was selected using Bayesian Information Criterion (BIC) (Neath & Cavanaugh, 2012) implemented in the program MEGA version 7 (Kumar, Stecher & Tamura, 2016). These results gave a best fit for the JTT + Γ (Jones, Taylor & Thornton, 1992) amino acid substitution model.

We executed three parallel MCMC runs simultaneously, each one was run for 30 × 10^6^ generations with four Markov chains (one cold and three heated chains). We used Tracer v1.6 (Rambaut & Drummond, 2013) for visualizing output parameters to ascertain stationarity and whether or not the duplicated runs had converged on the same mean likelihood. Nodes were considered supported if posterior probabilities (PP) > 0.95. Trees were visualized using FigTree v1.1 (Rambaut, 2009) available at http://tree.bio.ed.ac.uk/software/figtree/.

In order to establish the genetic distance among LAAOs obtained in this study with LAAOs corresponding to *Micrurus* species used in the phylogenetic analysis, a matrix distance from aligned *Micrurus* sequences was obtained in Mega Version 7 (Supplemental Information 2).

### Enzymatic characterization

LAAO activity was determined using the method of Kishimoto & Takahashi (2001). In brief, increasing doses (0.9 µg–3.0 µg) of MipLAAO (in 10 µL of water) were added to 90 µL of a reaction mixture (250 µM L-Leucine, 2 mM *o*-phenylenediamine, and 0.8 U/ml horseradish peroxidase) in 50 mM Tris pH 8.0 buffer. After incubation at 37 °C for 60 min, the reaction was stopped with 50 µL of 2 M H_2_SO_4_, and absorbances were recorded at 492 nm.

To determine the substrate specificity of MipLAAO, L-Leu was replaced with other L-amino acids (L-His, L-Ser, L-Arg, L-Ala, L-Trp, L-Glu, L-Cys, L-Lys, L-Tyr, L-Val, L-Ile, L-Thr), under standard assay conditions. The amount of MipLAAO in the reaction mixture was 1.5 µg. LAAO activity on L-Leu was also evaluated at different pH values (5.0–11.0).

### Antimicrobial assay

Antimicrobial assays were performed according to the National Committee for Clinical Laboratory Standards (Clinical and Laboratory Standards, 2009) using two methods: the first, agar diffusion assays, in which 5 µL (10 µg) of MipLAAO was added to media (Muller-Hinton medium) with a suspension of 1.5 ×10^6^ colony forming units (CFU)/mL of *E. coli* (ATCC 25922) or *S. aureus* (ATCC 25923) and incubated at 37 °C for 24 h. Sterile saline solution and chloramphenicol (10 µg; Phyto Technology Laboratories, Lenexa, KS, USA) were used as negative and positive controls, respectively. Diameters of the bacterial growth inhibition zones were measured (Vargas et al., 2013). Each assay was performed in triplicate.

The second method was the broth microdilution in 96-well plates (Clinical and Laboratory Standards, 2009). Inoculum suspensions of *S. aureus* cultures were prepared and adjusted to a density of 1.5 ×10^5^ CFU/mL. In each well, 50 µL of the bacterial suspension and 50 µL of MipLAAO (0.01–7.0 µg/mL) were mixed. Plates were incubated at 37 °C for 24 h, and the minimum inhibitory concentration (MIC) was defined as the lowest concentration of enzyme that prevented visible growth (absence of turbidity) in the broth. Assays were performed in triplicate. Sterile saline solution and chloramphenicol were used as controls.

### Statistical analysis

Results were expressed as mean ± SD. Analysis of variance (ANOVA- Kruskal-Wallis test) followed by Bonferroni post-test was employed to evaluate the statistical significance of data on LAAO activity, substrate preference, and pH effects. Differences were considered statistically significant when *p* < 0.05.

## Results

### Isolation, determination of molecular mass, cloning and amino acid sequencing of MipLAAO

**Figure 1:**
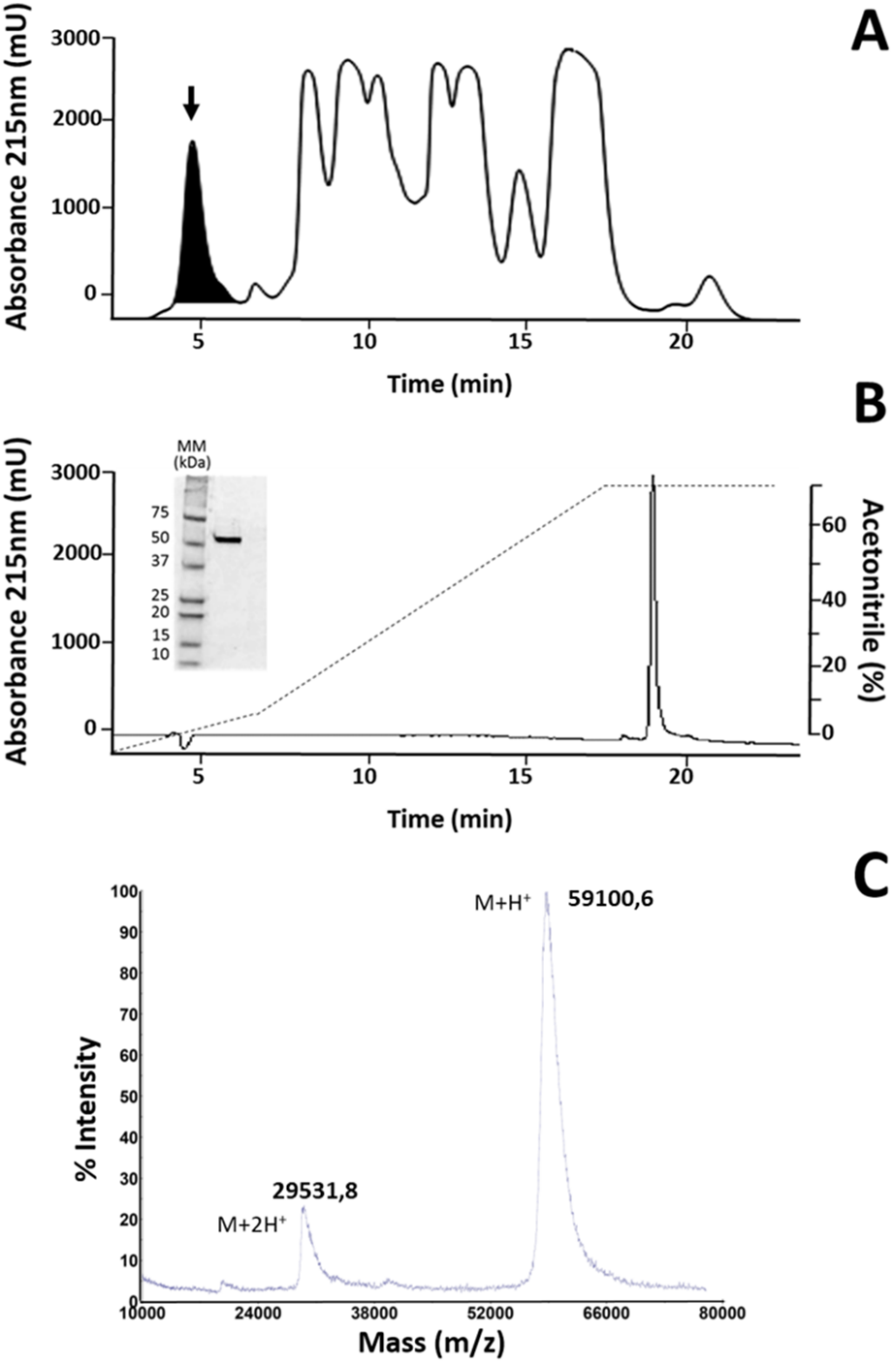
Isolation of MipLAAO from *M. mipartitus* venom. (A) SEC-HPLC separation of *M. mipartitus* venom (2 mg) on a Biosec S-200 column eluted at 0.7 mL/min with PBS. MipLAAO (shadowed area) was collected in the peak eluting at 4.58 min, indicated with an arrow. (B) Protein homogeneity was observed for RP-HPLC on *C*_18_ column eluted with a gradient of 0 to 70% solution B (Acetonitrile). The protein was analyzed by SDS-PAGE (15% gel) under reducing conditions (insert in B). MM, molecular mass standards, in kDa. (C) Molecular mass of the isolated MipLAAO by MALDI-TOF analysis.

The fraction identified as LAAO in *M. mipartitus* venom (Rey-Suárez et al., 2011), here named MipLAAO, was isolated using SEC-HPLC, with an retention time of 4.5 min, (Fig. 1A). This fraction corresponds to 6% of the chromatogram with a final yield of 100 µg per run. The protein eluted as a homogeneous peak by RP-HPLC analysis, at 19.6 min (Fig. 1B). SDS-PAGE of MipLAAO under reducing (Fig. 1B, insert), or non reducing conditions (not shown) both presented a single band, with a migration at ∼57 kDa, indicating that it is a monomeric protein.

**Figure 2:**
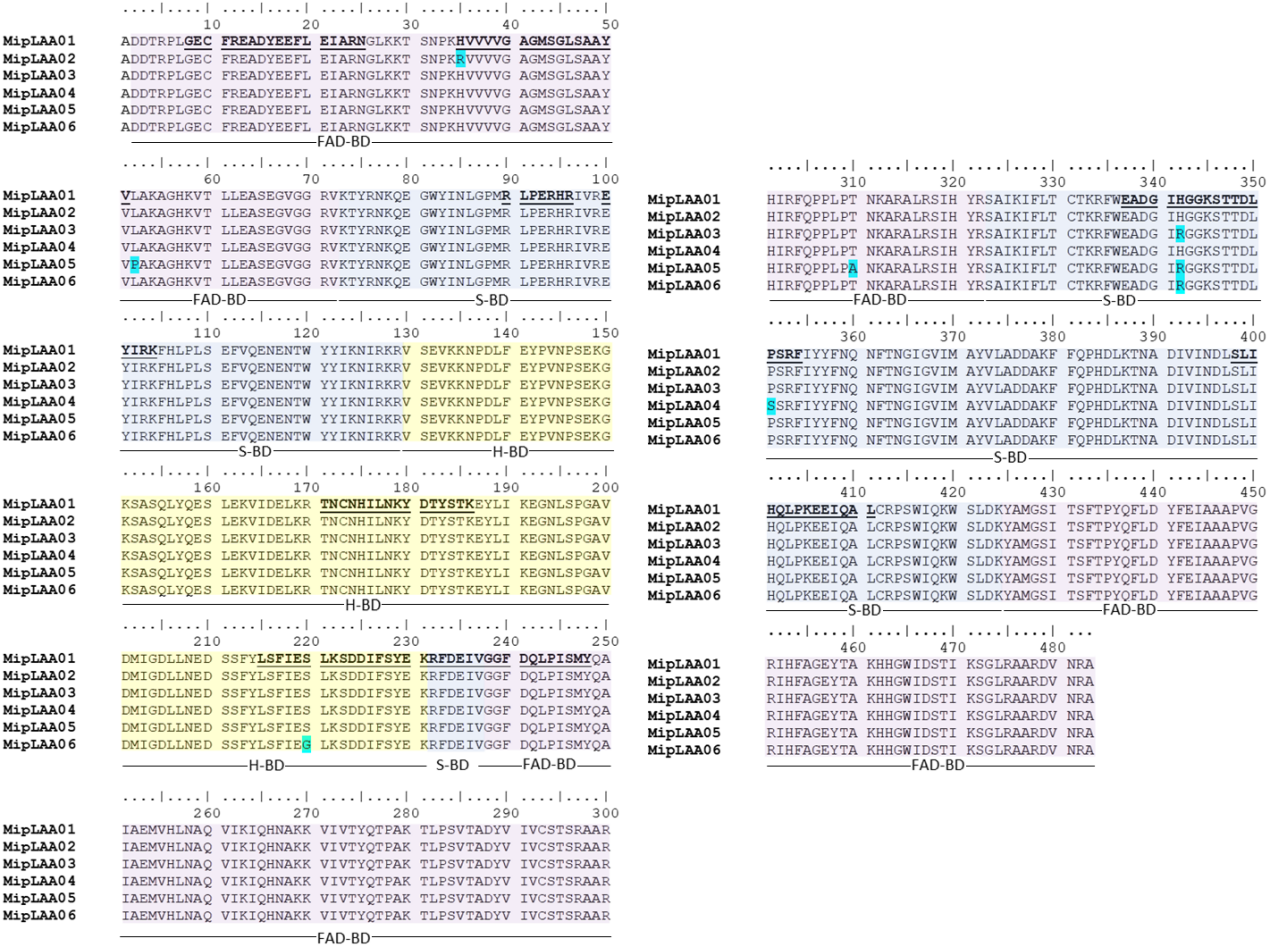
Amino acid sequences of six MipLAAO isoforms deduced from *M. mipartitus* venom gland cDNA sequences. Multiple alignment of 483 amino acids corresponding to the mature chains is shown. Polymorphic sites are highlighted in blue. Tryptic peptide sequences confirmed by MALDI-TOF/TOF MS are shown in bold and underlined in the MipLAAO-1 sequence. The three domains of LAAO are highlighted in colors: FAD-binding domain (FAD-BD in pink), substrate binding domain (S-BD in blue) and helical domain (H-BD in yellow). For reference to colors in this figure legend, the reader is referred to the web version of this article.

The cDNA obtained from the venom gland mRNA of *M. mipartitus* evidenced a product of ∼1,503 bp, corresponding to the expected molecular mass for the LAAO coding sequence. The primers amplified a DNA fragment, which was purified, ligated and used to transform *E. coli* cells. Eight positive clones, randomly selected, were sequenced and six coding sequences, including signal peptide, were obtained and designated as MipLAAO-1 to MipLAAO-6 (Fig. 2). These sequences were deposited in Genbank, with access codes (MH010800, MH010801, MH010802, MH010803, MH010804, MH010805).

Protein sequences deduced from the six cDNA clones consist of 501 amino acids, with a segment of 18 amino acids corresponding to signal peptide, and 483 amino acids corresponding to mature chain (Fig. 2). Few changes among the sequences were observed. MALDI-TOF/TOF peptide sequences obtained *de novo* matched the amino acid sequences deduced from cDNA (Fig. 2). These sequences evidenced high identity values compared to other LAAOs from elapid and viperid snakes (Supplemental Information 1). Highest identities were obtained against elapid LAAOs, especially from other *Micrurus*, the LAAOs of which have been predicted by transcriptomic studies, such as *M. spixi* LAAO (89% identity; Supplemental Information 1).

The observed isotope-averaged molecular mass of the isolated MipLAAO was 59,100.6 Da, by MALDI-TOF MS (Fig. 1C). The theoretical molecular masses predicted (https://web.expasy.org/peptide_mass/) from the nucleotide-sequenced clones MipLAAO-1 to -6 vary from 55,121.02 Da to 55,010.94 Da. These variations correspond to few amino acid substitutions among them (Fig. 2). The difference between these theoretical mass values and that determined for the isolated protein are suggestive of post-translational modification of the enzyme, for example glycosylation. MALDI-TOF/TOF peptide sequencing matched the predicted MipLAAO cDNA-deduced sequence, covering 56% (Fig. 2). In addition, the deduced amino acid sequences of MipLAAO isoforms predict these to be basic proteins, according to the Compute Mw/pI tool at https://web.expasy.org/compute_pi/, with pI values within the range of 8.85–8.93.

### Phylogenetic relationships

**Figure 3:**
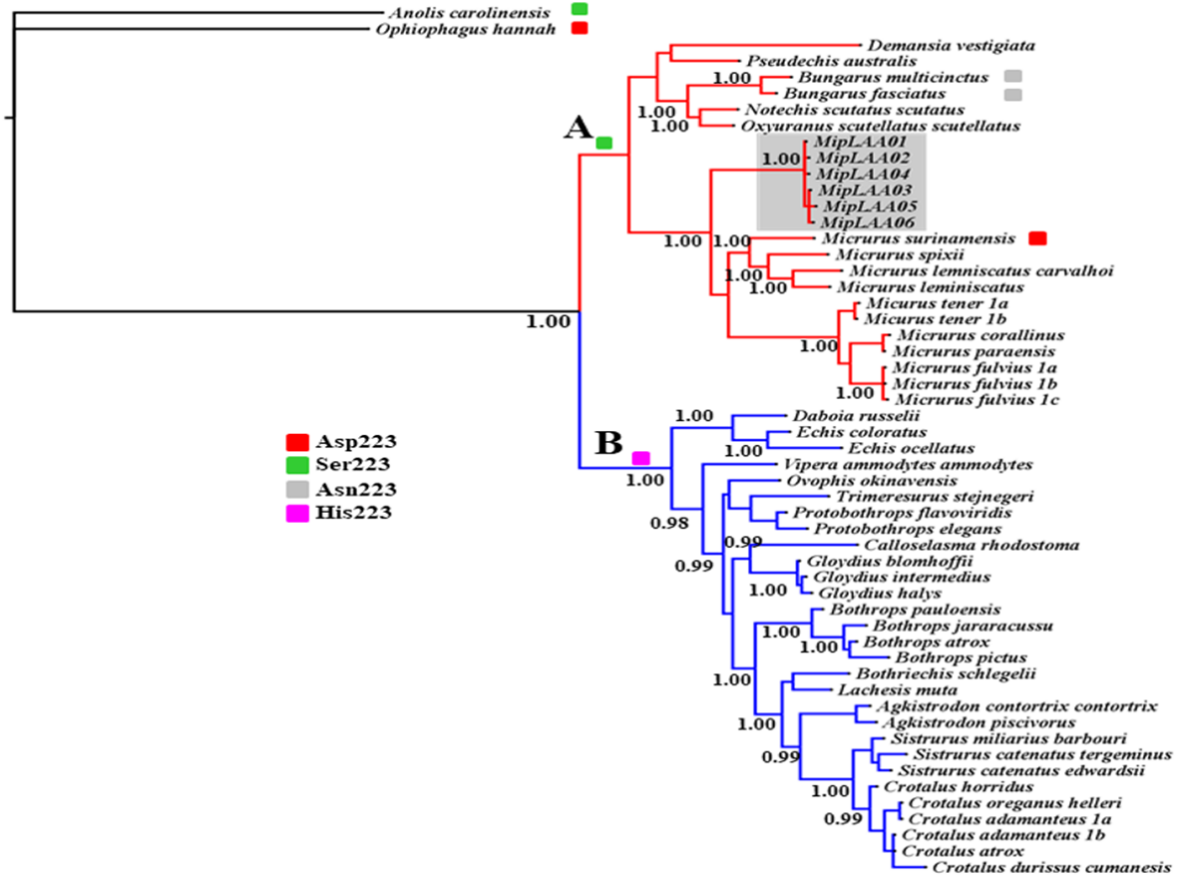
Bayesian phylogeny of relationship among LAAOs of venoms from the Elapidae (17 species) and Viperidae (30 species) families. Two distinct LAAO lineages are depicted in Clade A (Elapidae, in red) and Clade B (Viperidae, in blue). LAAO sequences obtained in this study are indicated with a gray shadow. The presence of Ser (green), Asp (red), Asn (gray) and His (pink) residues in the 223 position were shown. Support for each node is shown as posterior probability (PP). For reference to colors in this figure legend, the reader is referred to the web version of this article.

The deduced protein sequences of six MipLAAO isoforms obtained in this study and 48 other related LAAOs were aligned to infer phylogenetic relationships. Our analysis recovered a deep split of LAAOs into two lineages (Clades A and B, Fig. 3), separating all Elapidae family enzymes in Clade A from those of Viperidae family in Clade B, except for the King Cobra (*Ophiophagus hannah*) LAAO, which shares between 49.0% and 50.8% identity with other Elapidae LAAOs, and looped out with *Anolis carolinesis* LAAO (outgroup). This split, in clear agreement with snake taxonomy, is associated to differences in the amino acid residue at position 223, occupied by Ser in all LAAOs from clade A (with the exception of *M. surinamensis* and *Bungarus sp*., having Asp and Asn, respectively) or by His in all members of clade B.

Within Clade A (Elapidae family), all LAOOs from *Micrurus* conform a well-supported group (*PP* = 1.00) with genetic distance range between 12% with *M. spixii* and 18.9% with *M. tener* (Supplemental Information 2). MipLAAOs sequences here obtained present variations with other *Micrurus* LAAOs at positions 175 (His instead of Tyr), 301 (His instead of Arg) and 384 (His instead of Leu).

### Functional characterization of MipLAAO

**Figure 4:**
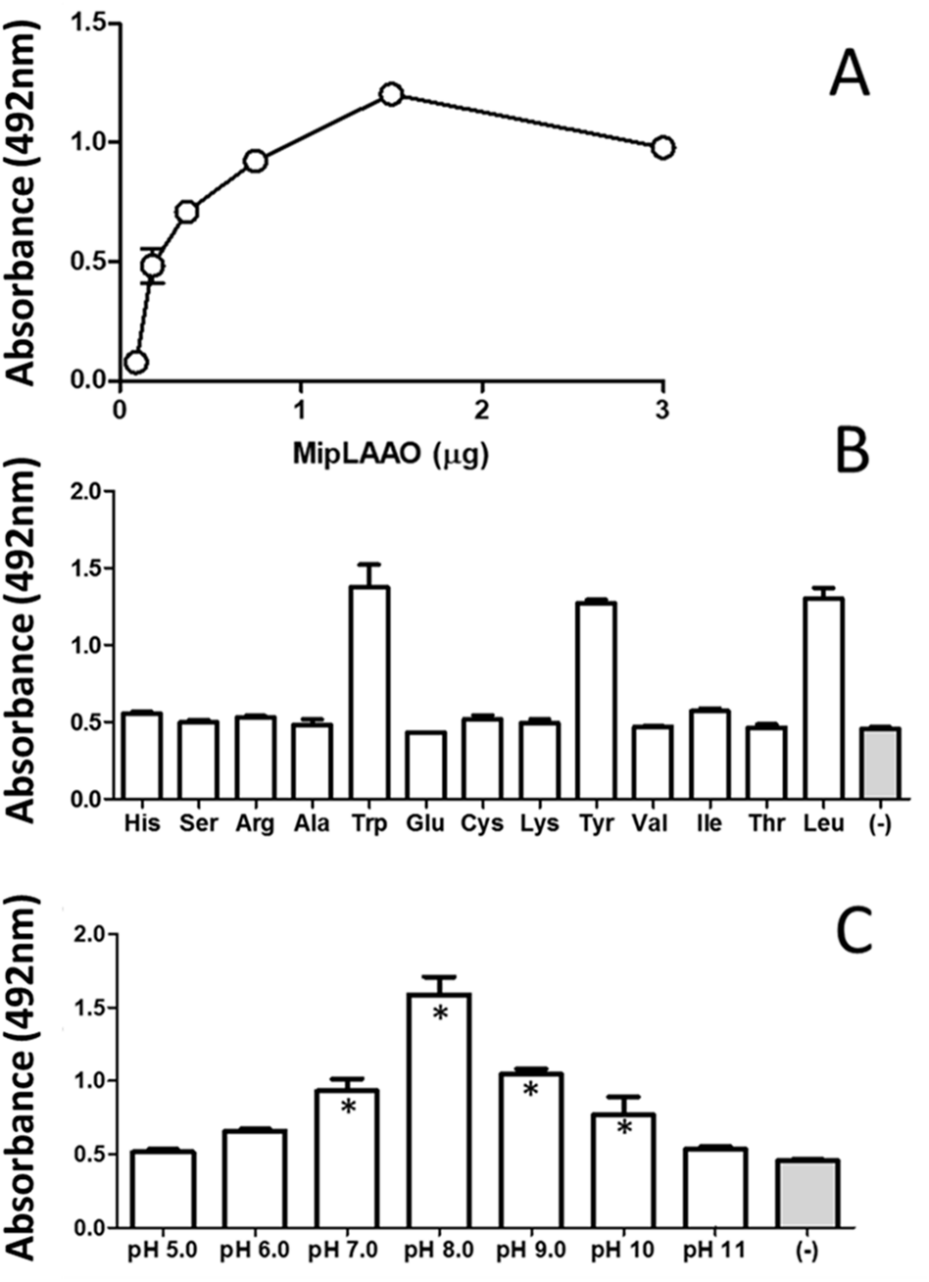
Functional characterization of MipLAAO. (A) Enzymatic activity on L-Leu. (B) Substrate preference: L-Leu was replaced with other L-amino acids (L-His, L-Ser, L-Arg, L-Ala, L- Trp, L-Glu, L-Cys, L-Lys, L-Tyr, L-Val, L-Ile, L-Thr) under standard assay conditions. (C) pH influence; the enzyme activity was tested at various pH conditions (5.0–11.0). Each point represents the mean ± SD (*n* = 3). * Represents statistical significant difference compared to the negative control.

MipLAAO was obtained in active form, presenting a conspicuous concentration-dependent enzymatic activity upon L-Leu (Fig. 4A). This enzyme only oxidized hydrophobic amino acids like L-Trp, L-Tyr, and L-Leu (Fig. 4B). Furthermore, the enzyme maintained its activity within the pH range 7.0–10.0, being optimal at pH 8.0 (Fig. 4C).

### Antibacterial activity of MipLAAO

MipLAAO exhibited antibacterial activity against *S. aureus*, but not against *E. coli*. Against the former, the enzyme produced a bacterial growth inhibition halo of 19.8 ± 0.6 mm, while chloramphenicol used as a control produced a halo of 12 ± 1 mm. The MIC against *S. aureus* evaluated by the broth microdilution method was 2 µg/mL.

## Discussion

LAAOs are widely found in snake venoms, both in Elapidae and Viperidae families. In *Micrurus*, this enzyme has been detected by proteomic or transcriptomic analyses in *M. corallinus* (Corrêa-Netto et al., 2011; Aird et al., 2017; Morais-Zani et al., 2018), *M. altirostris* (Corrêa-Netto et al., 2011), *M. fulvius* (Margres et al., 2013), *M. surinamensis* (Olamendi-Portugal et al., 2008; Aird et al., 2017), *M. mipartitus* (Rey-Suárez et al., 2011), *M. nigrocinctus* (Fernández et al., 2011), *M. lemniscatus*, *M. paraensis*, *M. spixii* (Aird et al., 2017), *M. clarki* (Lomonte et al., 2016), *M. tener* (Rokyta, Margres & Calvin, 2015), *M. mosquitensis* (Fernández et al., 2015), *M. alleni* (Fernández et al., 2015), and *M. tschudii* (Sanz et al., 2016).

The abundance of this enzyme in venoms varies among species, from traces (0.15%) as in *Naja oxiana* (Samel et al., 2008) to major proportions (25%) as in *Bungarus caeruleus* (More et al., 2010). In *Micrurus* venoms, it represents a low abundance component, with a range of 0.7–4.0% of the total proteins. Interestingly, the highest proportion of LAAO in this genus has thus far been found in *M. mipartitus* venom (4%; Rey-Suárez et al., 2011).

This study reports the first LAAO isolated and characterized from *Micrurus* venoms. MipLAAO is a basic protein with 483 amino acid residues. The enzyme was obtained in active form, showing substrate specificity for hydrophobic amino acids, and optimal catalytic activity at basic pH. Sequencing of cDNA clones obtained from the venom glands of a single individual, evidenced the presence of at least six isoforms, with few conservative differences among them. Similarly, transcriptomic analyses of *M. fulvius* (Margres et al., 2013), *M. tener* (Rokyta, Margres & Calvin, 2015), and *M. lemniscatus* (Aird et al., 2017) venom glands reported three, two, and two LAAO isoforms, respectively.

Structurally, the obtained MipLAAO sequences conserve the three well-known domains named FAD-binding domain (Asp2 to Val72, Gly238 to Arg322 and Tyr425 to Ala483), substrate binding domain (Lys73 to Arg129, Arg232 to Val237 and Ser323 to Lys424), and helical domain (Val130 to Lys231) (Feliciano et al., 2017). Few sequence variations were observed between the LAAO isoforms of *M. mipartitus* venom. In the FAD-binding domain, MipLAAO-2 has one substitution at residue His35, and MipLAAO-6 presents two, Leu52Pro and Thr329Ala. In the substrate binding domain, MipLAAO-4 displays one modification, Pro351Ser, and MipLAAO-3, 5, 6 all present the same change, His342Arg. Finally, in the helical domain, only MipLAAO-6 varied from the others, presenting the substitution Ser220Gly. The role of isoform sequence substitutions in the enzymatic activity and substrate preferences remains to be explored.

Amino acids involved in catalytic activity of the enzyme (Arg90, Tyr372, Gly464, Ile430, Phe227, and Lys326) are conserved in the sequences obtained for *M. mipartitus* LAAO, with the exception of residue 374 that presents a conservative Ile/Leu substitution. This amino acid is of key importance given that it participates in the hydrophobic interactions that are formed with the side chains of the substrate in the catalytic mechanisms (Izidoro et al., 2014).

As for almost all Elapidae LAAOs, MipLAAO presents Ser at position 223, which is occupied by His in LAAOs from Viperidae. His223 has been shown to play an important role in the enzymatic reaction during the binding of substrate to the catalytic site (Moustafa et al., 2006). However, His223 is substituted by Ser in almost all sequences of venom LAAOs from Elapidae, except in *M. surinamensis* (Asp223), *B.multicinctus,* and *B. multifasciatus* (Asn223). Chen et al. (2012) observed that His223 was present in all viperid LAAOs, Ser223 in most of elapid LAAOs, while Asn223 is present in krait LAAOs, and Asp223 is found in king cobra LAAO. These authors suggested that the different residues at position 223 may regulate substrate specificities of LAAOs by expanding the substrate-binding pocket and reducing steric repulsion.

MipLAAO has three potential N-glycosylation sites at Asn145, Asn194, and Asn361, indicating that it possibly presents this post-translational modification, in similarity with other venom LAAOs (Chen et al., 2012). Furthermore, this could explain the difference between the calculated and observed molecular mass values. It has been suggested that carbohydrates in LAAOs could contribute to cytotoxicity by mediating the binding of the enzyme to the cell surface and local accumulation of H_2_O_2_ (Geyer et al., 2001).

Similar to other svLAAOs (Ponnudurai, Chung & Tan, 1994; Sakurai et al., 2001; Vargas et al., 2013; Zhong et al., 2009; Jin et al., 2007), MipLAAO oxidized a variety of L-amino acids, especially the hydrophobic L-Trp, L-Tyr and L-Leu, indicating its optical isomer selectivity. In contrast, positively charged amino acids such as L-Lys and L-Arg present unfavorable electrostatic interactions with the catalytic site of the enzyme and are not oxidized (Moustafa et al., 2006).

Snake venom LAAOs exhibit wide ranges of pI, from acidic (Alves et al., 2008; Tõnismägi et al., 2006; Toyama et al., 2006; Izidoro et al., 2006; Rodrigues et al., 2009; Okubo et al., 2012; Vargas Muñoz et al., 2014) to basic (Lu et al., 2018; Zhang & Wei, 2007; Zhang et al., 2004; Vargas et al., 2013). According to its sequence, MipLAAO is predicted to be a basic protein, with a theoretical pI of 8.9. Differences in charge density may influence the pharmacological activities of LAAOs (Izidoro et al., 2014).

Many snake venom LAAOs have been shown to be bactericidal (Toyama et al., 2006; Stábeli et al., 2007; Rodrigues et al., 2009; Zhong et al., 2009; Lee et al., 2011; Vargas et al., 2013; Vargas Muñoz et al., 2014; Lazo et al., 2017). Bacteria inhibited by these enzymes include the Gram-positives *Bacillus dysenteriae, B. megatherium, B. subtilis, S. aureus* and *S. mutans,* and Gram-negative bacteria such as *Aeromonas* sp., *E. coli, Pseudomonas aeruginosa, Salmonella typhimurium, Acinetobacter baumannii* and *Xanthomonas axonopodis pv passiflorae* (Lee et al., 2011). According to literature, the most likely mode of action involved in the bactericidal activity of LAAOs involves oxidative stress in the target cell caused by hydrogen peroxide, triggering disorganization of the plasma membrane and cytoplasm, and consequent cell death (Toyama et al., 2006; Souza et al., 1999; Izidoro et al., 2014). These activities can be inhibited by the addition of catalase and other H_2_O_2_ scavengers (Torii, Naito & Tsuruo, 1997; Tempone et al., 2001; Suhr & Kim, 1999; Zhang et al., 2004). However, according to several studies, the role played by hydrogen peroxide in the biological activities of LAAOs is uncertain. It is likely that there are other mechanisms involved in their pharmacological and toxicological effects (Suhr & Kim, 1999; Zhang et al., 2004; Izidoro et al., 2014).

Lee et al. (2011) suggest that binding to bacteria is important for the activity of LAAO, as the concentration of H_2_O_2_ generated by the enzyme is not sufficient to kill bacteria. It was argued that binding of LAAO to the bacteria enables production of highly localized concentrations of H_2_O_2_ in or near the binding sites that will be sufficiently potent to kill the bacteria. This also explains why a very small amount of LAAO could effectively inhibit bacteria growth, as the MIC of King cobra LAAO against *S. aureus* was as low as 0.78 µg/mL (Lee et al., 2011). The interaction of LAAO with different cell membranes was demonstrated by Abdelkafi-Koubaa et al. (2016) in a study with CC-LAAO from *Cerastes cerastes* venom.

Present results showed that MipLAAO was effective against the Gram-positive *S. aureus*, with a low MIC value of 2 µg/mL, whereas the Gram-negative *E. coli* was not affected. This is in agreement with the selectivity of antibacterial action described for LAAOs of *B. schlegelii* (Vargas Muñoz et al., 2014), *O. hannah* (Lee et al., 2011), *C. durissus cumanensis* (Vargas et al., 2013), *D. russellii siamensis* (Zhong et al., 2009), *C. durissus cascavella* (Toyama et al., 2006), and *T. mucrosquamatus* (Wei et al., 2003) against gram positive bacteria. On the other hand, LAAOs from *B. pauloensis* (Rodrigues et al., 2009), *V. labetina* (Tõnismägi et al., 2006), *N. oxiana* (Samel et al., 2008) and *P. australis* (Stiles, Sexton & Weinstein, 1991) were more active against Gram-negative than Gram-positive bacteria, and LAAOs from *A. halys*, *B. alternatus*, and *B. moojeni* inhibited both Gram-positive and Gram-negative bacteria. These differences in the selectivity of the antibacterial action of LAAOs are presumably due to differences in their binding to bacteria.

## Conclusions

MipLAAO, the first LAAO characterized from a coral snake venom, is a basic protein with 483 amino acid residues. The enzyme was obtained in active form, showing substrate specificity for hydrophobic amino acids, and optimal catalytic activity at basic pH. It showed a significant antimicrobial effect against *S. aureus*, a clinically relevant Gram-positive bacterium. It will be of interest to explore its potential applications as antimicrobial agent in future studies.

##  Supplemental Information

10.7717/peerj.4924/supp-1 Supplemental Information 1 Supplementary Material 1 Homologous sequences svLAAOS found in uniprot.

10.7717/peerj.4924/supp-2 Supplemental Information 2 Supplementary Material 2 Net divergences using JTT + G amino acid evolution model for LAAOs of *Micrurus* species.

## Additional Information and Declarations

### DNA Deposition

The following information was supplied regarding the deposition of DNA sequences: These sequences were deposited in Genbank, with accession numbers: MH010800, MH010801, MH010802, MH010803, MH010804, MH010805.

### Data Availability

The following information was supplied regarding data availability: The raw data are provided in the Figures in the article.
