# Supplementary material for: MipLAAO, a new L-amino acid oxidase from the redtail coral snake Micrurus mipartitus
Source: PeerJ. 2018 Jun 8;6:e4924. doi: 10.7717/peerj.4924 (PMC5995095; doi:10.7717/peerj.4924)
Supplement: Supplemental Information 1 — Homologous sequences svLAAOS found in uniprot. [file peerj-06-4924-s001.doc]

**Supplemental S1**: svLAAO sequences homologous to MmipLAAO^*^ found in UniProt

| **No.** | **Accession** | **Species** | **E-value** | **Score** | **Ident. %** |
| --- | --- | --- | --- | --- | --- |
| 1 | MH010800 | *M. mipartitus* MmipLAA01 | 0.0 | 2.610 | 100.0 |
| 2 | MH010801 | *M. mipartitus* MmipLAA04 | 0.0 | 2.604 | 99.8 |
| 3 | MH010802 | *M. mipartitus* MmipLAA02 | 0.0 | 2.602 | 99.8 |
| 4 | MH010803 | *M. mipartitus* MmipLAA03 | 0.0 | 2.602 | 99.7 |
| 5 | MH010804 | *M. mipartitus* MmipLAA06 | 0.0 | 2.598 | 99.6 |
| 6 | MH010805 | *M. mipartitus* MmipLAA05 | 0.0 | 2.590 | 99.4 |
| 7 | DN100626 | *Micrurus spixii* | 0.0 | 2.322 | 89.0 |
| 8 | DN120559 | *Micrurus lemniscatus* | 0.0 | 2.285 | 89.0 |
| 9 | DN77443 | *Micrurus surinamensis* | 0.0 | 2.258 | 87.2 |
| 10 | DN67860 | *Micrurus lemniscatus carvalhoi* | 0.0 | 2.256 | 87.6 |
| 11 | DN81361 | *Micrurus paraensis* | 0.0 | 2.232 | 85.5 |
| 12 | DN51373 | *Micrurus corallinus* | 0.0 | 2.227 | 85.3 |
| 13 | A0A194ARE6 | *Micrurus tener 1b* | 0.0 | 2.216 | 84.8 |
| 14 | A0A194ASA8 | *Micrurus tener 1a* | 0.0 | 2.212 | 84.6 |
| 15 | U3EPI5 | *Micrurus fulvius 1c* | 0.0 | 2.201 | 84.4 |
| 16 | U3FYQ2 | *Micrurus fulvius 1a* | 0.0 | 2.200 | 84.4 |
| 17 | U3FYQ2B | *Micrurus fulvius 1b* | 0.0 | 2.200 | 84.4 |
| 18 | Q4JHE2 | *Notechis scutatus scutatus* | 0.0 | 2.104 | 83.2 |
| 19 | Q4JHE3 | *Oxyuranus scutellatus scutellatus* | 0.0 | 2.104 | 83.4 |
| 20 | Q4JHE1 | *Pseudechis australis* | 0.0 | 2.095 | 82.8 |
| 21 | A8QL51 | *Bungarus multicinctus* | 0.0 | 2.056 | 80.8 |
| 22 | A8QL52 | *Bungarus fasciatus* | 0.0 | 2.044 | 81.0 |
| 23 | T2HRS5 | *Protobothrops flavoviridis* | 0.0 | 1.996 | 78.7 |
| 24 | Q90W54 | *Gloydius blomhoffii* | 0.0 | 1.983 | 77.4 |
| 25 | A0A068EPZ2 | *Gloydius intermedius* | 0.0 | 1.983 | 77.2 |
| 26 | A0A0S1LJ33 | *Bothrops atrox* | 0.0 | 1.977 | 77.0 |
| 27 | Q6STF1 | *Gloydius halys* | 0.0 | 1.973 | 77.2 |
| 28 | B5AR80 | *Bothrops pauloensis* | 0.0 | 1.970 | 77.0 |
| 29 | T2HQ57 | *Ovophis okinavensis* | 0.0 | 1.965 | 77.2 |
| 30 | A0A077L6L4 | *Protobothrops elegans* | 0.0 | 1.954 | 77.4 |
| 31 | A6MFL0 | *Demansia vestigiata* | 0.0 | 1.953 | 77.5 |
| 32 | G8XQX1 | *Daboia russelii* | 0.0 | 1.949 | 77.0 |
| 33 | A0A0A1WCY6 | *Echis coloratus* | 0.0 | 1.941 | 76.8 |
| 34 | X2L4E2 | *Bothrops pictus* | 0.0 | 1.940 | 75.6 |
| 35 | P0DI84 | *Vipera ammodytes ammodytes* | 0.0 | 1.938 | 76.7 |
| 36 | J7H670 | *Lachesis muta* | 0.0 | 1.938 | 75.8 |
| 37 | A0A194APS7 | *Sistrurus catenatus tergeminus* | 0.0 | 1.931 | 75.5 |
| 38 | Q6WP39 | *Trimeresurus stejnegeri* | 0.0 | 1.930 | 75.8 |
| 39 | Q6TGQ9 | *Bothrops jararacussu* | 0.0 | 1.929 | 75.6 |
| 40 | K9N7B7 | *Crotalus durissus cumanesis* | 0.0 | 1.924 | 76.1 |
| 41 | P81382 | *Calloselasma rhodostoma* | 0.0 | 1.917 | 75.4 |
| 42 | A0A0K8RYS7 | *Crotalus horridus* | 0.0 | 1.917 | 75.7 |
| 43 | B5U6Y8 | *Echis ocellatus* | 0.0 | 1.915 | 74.9 |
| 44 | A0A1941S07 | *Sistrurus miliarius barbouri* | 0.0 | 1.904 | 74.8 |
| 45 | T1DP54 | *Crotalus oreganus helleri* | 0.0 | 1.903 | 75.1 |
| 46 | O93364 | *Crotalus adamanteus 1a* | 0.0 | 1.903 | 75.1 |
| 47 | A0A0F7Z0X7 | *Crotalus adamanteus 1b* | 0.0 | 1.903 | 75.5 |
| 48 | P56742 | *Crotalus atrox* | 0.0 | 1.903 | 75.5 |
| 49 | B0VXW0 | *Sistrurus catenatus edwardsii* | 0.0 | 1.896 | 74.6 |
| 50 | A0A024BTN9 | *Bothriechis schlegelii* | 0.0 | 1.895 | 75.2 |
| 51 | A0A194APF4 | *Agkistrodon contortrix* | 0.0 | 1.890 | 74.1 |
| 52 | A0A194APV2 | *Agkistrodon piscivorus* | 0.0 | 1.887 | 73.9 |
| 53 | P81383 | *Ophiophagus hannah* | 1.6e-162 | 1.240 | 50.8 |
| 54 | R4GAU3 | *Anolis carolinensis* | 1.2e-162 | 1.242 | 50.3 |

* Identities calculated in comparison to isoform *M. mipartitus* MmipLAA01
