# Supplementary material for: MipLAAO, a new L-amino acid oxidase from the redtail coral snake Micrurus mipartitus
Source: PeerJ. 2018 Jun 8;6:e4924. doi: 10.7717/peerj.4924 (PMC5995095; doi:10.7717/peerj.4924)
Supplement: Supplemental Information 2 — Net divergences using JTT + G amino acid evolution model for LAAOs of Micrurus species. [file peerj-06-4924-s002.doc]

**Supplemental S2**: Net divergences using JTT+G amino acid evolution model for LAAOs of *Micruru*s species (below diagonal). Standard errors are shown above diagonal. In gray, Genetic differentiation among MipLAAO isoforms. In Blue, *Micrurus* species with lower genetic difference compared to MipLAAOs,

|  |  | **1** | **2** | **3** | **4** | **5** | **6** | **7** | **8** | **9** | **10** | **11** | **12** | **13** | **14** | **15** | **16** | **17** |
| --- | --- | --- | --- | --- | --- | --- | --- | --- | --- | --- | --- | --- | --- | --- | --- | --- | --- | --- |
| **1** | **MipLAAO-1** |  | 0.002 | 0.002 | 0.002 | 0.003 | 0.004 | 0.017 | 0.018 | 0.020 | 0.019 | 0.021 | 0.021 | 0.022 | 0.022 | 0.022 | 0.022 | 0.022 |
| **2** | **MipLAAO-4** | 0.002 |  | 0.003 | 0.003 | 0.003 | 0.004 | 0.018 | 0.018 | 0.020 | 0.019 | 0.021 | 0.022 | 0.022 | 0.022 | 0.022 | 0.022 | 0.022 |
| **3** | **MipLAAO-2** | 0.002 | 0.004 |  | 0.003 | 0.003 | 0.004 | 0.018 | 0.018 | 0.020 | 0.020 | 0.021 | 0.022 | 0.022 | 0.022 | 0.022 | 0.022 | 0.022 |
| **4** | **MipLAAO-3** | 0.002 | 0.004 | 0.004 |  | 0.002 | 0.003 | 0.017 | 0.017 | 0.020 | 0.019 | 0.021 | 0.022 | 0.022 | 0.022 | 0.022 | 0.021 | 0.021 |
| **5** | **MipLAAO-6** | 0.004 | 0.006 | 0.006 | 0.002 |  | 0.003 | 0.017 | 0.018 | 0.020 | 0.019 | 0.022 | 0.022 | 0.022 | 0.022 | 0.022 | 0.022 | 0.022 |
| **6** | **MipLAAO-5** | 0.006 | 0.008 | 0.008 | 0.004 | 0.006 |  | 0.017 | 0.018 | 0.020 | 0.019 | 0.022 | 0.022 | 0.022 | 0.022 | 0.022 | 0.022 | 0.022 |
| **7** | ***M. spixii*** | 0.123 | 0.125 | 0.125 | 0.120 | 0.123 | 0.123 |  | 0.014 | 0.016 | 0.015 | 0.022 | 0.022 | 0.020 | 0.020 | 0.021 | 0.021 | 0.021 |
| **8** | ***M. lemniscatus*** | 0.128 | 0.130 | 0.130 | 0.125 | 0.128 | 0.128 | 0.092 |  | 0.016 | 0.012 | 0.021 | 0.020 | 0.020 | 0.020 | 0.020 | 0.019 | 0.019 |
| **9** | ***M. surinamensis*** | 0.148 | 0.150 | 0.150 | 0.150 | 0.153 | 0.153 | 0.107 | 0.104 |  | 0.017 | 0.021 | 0.021 | 0.019 | 0.019 | 0.019 | 0.020 | 0.020 |
| **10** | ***M.l. carvalhoi*** | 0.141 | 0.143 | 0.143 | 0.138 | 0.141 | 0.141 | 0.097 | 0.064 | 0.116 |  | 0.020 | 0.020 | 0.019 | 0.019 | 0.020 | 0.020 | 0.020 |
| **11** | ***M. paraensis*** | 0.171 | 0.174 | 0.174 | 0.174 | 0.177 | 0.177 | 0.171 | 0.165 | 0.175 | 0.155 |  | 0.003 | 0.009 | 0.009 | 0.010 | 0.010 | 0.010 |
| **12** | ***M. corallinus*** | 0.174 | 0.177 | 0.177 | 0.177 | 0.180 | 0.180 | 0.171 | 0.160 | 0.169 | 0.155 | 0.004 |  | 0.009 | 0.010 | 0.011 | 0.010 | 0.010 |
| **13** | ***M. tener 1b*** | 0.181 | 0.183 | 0.183 | 0.183 | 0.186 | 0.186 | 0.159 | 0.160 | 0.158 | 0.144 | 0.037 | 0.041 |  | 0.002 | 0.009 | 0.009 | 0.009 |
| **14** | ***M. tener 1a*** | 0.183 | 0.186 | 0.186 | 0.186 | 0.189 | 0.189 | 0.162 | 0.162 | 0.160 | 0.146 | 0.039 | 0.043 | 0.002 |  | 0.009 | 0.009 | 0.009 |
| **15** | ***M. fulvius 1c*** | 0.183 | 0.186 | 0.186 | 0.180 | 0.183 | 0.183 | 0.168 | 0.160 | 0.167 | 0.153 | 0.046 | 0.050 | 0.043 | 0.040 |  | 0.002 | 0.002 |
| **16** | ***M. fulvius 1a*** | 0.182 | 0.185 | 0.185 | 0.180 | 0.183 | 0.183 | 0.168 | 0.159 | 0.168 | 0.153 | 0.046 | 0.050 | 0.040 | 0.038 | 0.002 |  | 0.000 |
| **17** | ***M. fulvius 1b*** | 0.182 | 0.185 | 0.185 | 0.180 | 0.183 | 0.183 | 0.168 | 0.159 | 0.168 | 0.153 | 0.046 | 0.050 | 0.040 | 0.038 | 0.002 | 0.000 |  |
